# Equitable imagery in global health: a qualitative study examining how to create agency, share power and build partnership

**DOI:** 10.1136/bmjgh-2026-024519

**Published:** 2026-07-13

**Authors:** Alexandra M Cardoso Pinto, Anastasia Koch, Sipho Dlamini, Esmita Charani, Ed Young

**Affiliations:** 1 School of Public Health, Imperial College London, London, UK; 2 Oxford Clinical Academic Graduate School, University of Oxford, Oxford, UK; 3 Molecular Mycobacteriology Research Unit, University of Cape Town, Rondebosch, South Africa; 4 Eh!woza, Cape Town, Khayelitsha, South Africa; 5 Department of Pathology, Institute of Infectious Disease and Molecular Medicine, Rondrbosch, South Africa; 6 Division of Infectious Diseases and HIV Medicine, Department of Medicine, Groote Schuur Hospital, University of Cape Town, Cape Town, South Africa

**Keywords:** Global Health, Health education and promotion, Paediatrics, Interdisciplinary Research, Social Stigma

## Abstract

**Objectives:**

To explore how ethical image-making practices in global health are shaped by issues of agency, power and partnership across the planning, creation and dissemination of imagery and to examine stakeholder perspectives on applying a previously published ethical imagery framework.

**Setting:**

International qualitative study involving stakeholders engaged in the commissioning, production and dissemination of Global Health imagery across multiple countries, combined with survey data from young people participating in a photography and ethics programme in Khayelitsha, Cape Town, South Africa.

**Participants:**

19 stakeholders involved in Global Health imagery participated in semi-structured interviews. Additionally, 18 young people aged 15–17 years participating in an 8-month photography training programme completed a survey.

**Primary and secondary outcome measures:**

The primary outcome was qualitative exploration of stakeholder perspectives on ethical imagery practices across planning and commissioning, image creation and dissemination. Secondary outcomes included survey findings from young people regarding priorities for ethical representation in global health imagery.

**Results:**

Thematic analysis identified three interconnected themes: agency and autonomy, power imbalances and inequity and collaborative partnerships. Participants highlighted that ethical concerns arise throughout the image-making process and are shaped by structural inequities including who determines what is photographed, how it is framed and where it is disseminated. Meaningful consent, inclusive representation, community involvement and shared decision-making were identified as central to ethical practice. Participants viewed the framework as a useful tool for ethical reflection but emphasised that its effectiveness depends on training, institutional commitment and accountability mechanisms. Survey findings reinforced the importance of dignity, representation and participant involvement in decisions about imagery.

**Conclusions:**

Global Health imagery requires approaches that extend beyond technical guidance to address power imbalances and promote meaningful partnership. Frameworks should support dialogue, reflexivity and shared accountability rather than function as prescriptive rulebooks. Institutions, image-makers and communities should work collaboratively to ensure imagery reinforces dignity, agency, equity and solidarity in Global Health.

**WHAT IS ALREADY KNOWN ON THIS TOPIC**

**WHAT THIS STUDY ADDS**

- This work incorporates perspectives from photographers, researchers, non-governmental organisations and young people from a variety of settings.
- It explores ethical challenges across three stages of global health imagery: planning and commissioning, image creation and dissemination.
- The work identifies three themes across these stages as central factors shaping ethical image-making: agency, power imbalances and partnerships.

**HOW THIS STUDY MIGHT AFFECT RESEARCH, PRACTICE OR POLICY**

- All institutions involved in Global Health research and communication should integrate ethical governance, including reflection, of imagery into commissioning processes, training and organisational policy.
- Strengthening partnerships with communities and artists can support imagery that reinforces dignity, agency and equity.
- More equitable practices include co-created briefs, community consultation and shared review before publication.

## Introduction

 Visual communication plays a central role in Global Health across reports, publications, teaching materials, campaigns, policy briefs and public engagement. Beyond complementing written narratives, imagery functions as a persuasive medium that conveys complex ideas in immediate, accessible and emotionally resonant ways.^1 2^ Images are not neutral: they construct narratives, shape public perception and opinion and influence decision-making, with implications for funding priorities and policy directions.^3^,^5^ By evoking empathy, urgency or solidarity, imagery can mobilise support for Global Health interventions.^6 7^ However, it can also reinforce inequities when it perpetuates stereotypes, relies on sensationalism or obscures the agency of represented communities.^47^,^9^

The use of imagery in Global Health must be situated within broader critiques of post-colonial dynamics and development communication.^10^ Decision-making and representation are often dominated by organisations and media based in high-income countries,^11 12^ reinforcing narratives that position these actors as providers of expertise and solutions. In contrast, individuals and communities from low- and middle-income countries (LMICs) are frequently depicted as passive recipients of aid, with limited agency.^8^ Such asymmetrical portrayals perpetuate power imbalances and shape the priorities of donors, policymakers and Global Health institutions, reflecting broader inequities in Global Health practice and governance.^13^,^15^

A review of Global Health reports on infectious diseases by Charani *et al* (2023) demonstrated that imagery often reinforces harmful stereotypes in LMICs, with women and children of colour commonly portrayed as helpless and dependent on external support. These representations undermine dignity, erase agency and prioritise narratives of vulnerability over self-determination and solidarity. The framework proposed in that study has since been adopted by other scholars and groups as a tool for critically evaluating visual communication, underscoring its relevance and applicability.^16^ Building on this work, we sought perspectives from a broader range of stakeholders involved in the commissioning, production and dissemination of Global Health imagery. Additionally, through workshops with young media producers and residents of a township in Cape Town, South Africa who may be represented in such imagery, this study aimed to foreground lived experience in discussions of ethical practice.

## Methods

This work was conducted in partnership with Eh!woza, a Khayelitsha (Cape Town, South Africa) based non-profit organisation that engages young people from communities heavily affected by health and social inequalities through creative, research-informed workshops. Participants produce media, such as short documentaries, to explore and communicate the personal and social dimensions of their chosen Global Health issue (for instance, a particular infectious disease, the impact of climate change or any others), thereby promoting public awareness, stimulating dialogue and reducing stigma.^17 18^

### Participant recruitment

Potential participants were identified through previous engagement with international coalitions, particularly non-governmental organisation (NGO) representatives and photographers who had expressed interest during earlier presentations of the original framework to guide practice (Charani *et al*, 2023). To ensure diverse perspectives and representation of key stakeholders, invitations were also extended to experts affiliated with collaborating academic institutions, including healthcare workers, researchers, health advocates, artists and curators. Efforts were made to ensure variation across gender, ethnicity, age and geography. Young people working with Eh!woza, who were part of an 8-month photography training programme and had attended workshops on ethics in imagery, were also invited to participate in a written survey for this project. Young people working with Eh!woza were recruited through a partnership with an NGO that provides afterschool tutoring in Khayelitsha. After a workshop explaining the project, young people were invited to apply for the photography programme through a short application form. Applications were reviewed by a committee within Eh!woza, including young people living in Khayelitsha, and were reviewed for commitment and an interest in telling stories, rather than academic acumen.

### Data collection

This study employed an exploratory mixed-methods approach that combined semi-structured interviews and survey data. While the primary methodology was qualitative, the interview findings were supplemented by qualitative and descriptive survey data collected from young participants involved in the Eh!woza photography programme. These survey responses provided additional contextual insights from young people and contributed to the triangulation of interview findings.

This study used qualitative semi-structured interviews with participants recruited using purposive and snowball sampling strategies, with recruitment continuing until no substantially new concepts were identified from the data. Interviews were held online via Microsoft Teams and lasted 30–60 min. The interview guide (see online supplemental material) was developed from the four domains of the previously published framework and refined through iterative discussion among the multidisciplinary research team.^8^ The data collection tools were piloted with individuals who met the study eligibility criteria, including collaborators affiliated with Eh!woza, researchers and other stakeholders involved in Global Health imagery, to assess clarity and structure prior to data collection.

All interviews were recorded and transcribed using Otter AI, and the transcriptions were reviewed for errors. The interviews focused on the four core themes of the previously published framework: relevance, dignity, consent and representation.^8^ These themes were examined in the context of practical application, key barriers to implementation and broader implications for equity in Global Health. Emphasis was placed on the use of imagery involving vulnerable populations, including children and young people.

Additionally, young people who participated in the Eh!woza photography training programme were invited to complete a piloted survey comprising 11 questions and to share views on Global Health imagery and what they would like to change about it, using a mixture of Likert-scale and free-text questions.

### Data storage and protection

Data were stored on secure servers at the University of Cape Town and Imperial College London. Interview recordings were deleted on transcription, and transcripts anonymised. Data collection from young people was conducted through handwritten surveys, without any identifiable information.

### Data analysis

Thematic analysis was conducted using an inductive approach to identify patterns and emerging concepts within the data, using NVivo V.14.0. Transcripts were read iteratively, with initial codes generated and subsequently organised into broader themes aligned with or extending beyond the original framework’s four domains. Attention was paid to suggestions for refining the framework and any ethical considerations specific to vulnerable groups, such as children and young people. To support rigour of the analysis, we used iterative coding and reflexive discussion within the multidisciplinary research team as themes emerged throughout the analysis stage. The manuscript was informed by the Standards for Reporting Qualitative Research (SRQR).^19^

### Reflexivity statement

With a team based in the UK and South Africa, we are mindful of the power dynamics that shape Global Health research, particularly in cross-continental collaboration. We recognise the privilege held by those of us working from high-income settings and the potential influence this may have on how findings are interpreted and represented. Working closely with Eh!woza ensured the research was informed by local knowledge, lived experiences and cultural context. Our team is also multidisciplinary, with members from a variety of healthcare, academic and image-making and conceptual arts backgrounds.

## Results

A total of 19 interviews were completed (table 1), and 18 survey responses were collected (online supplemental table S1). Survey respondents were all Eh!woza learners, aged 15–17 years and living in Khayelitsha.

**Table 1:** Demographics for interview (n=19) and survey (n=18) participants

Participant demographic	Interview participants n (%)	Survey participants n (%)
Profession		
Academic	6 (31.6)	N/A
Artist (including photographers)	8 (47.4)	
Healthcare worker	2 (10.5)	
Non-governmental organisation (non-clinical) worker and/or marketing	4 (26.3)	
Gender		
Female	9 (47.4)	10 (55.6)
Male	10 (52.6)	8 (44.4)
Other	0	0
Base continent		
Africa	8 (42.1)	18 (100)
Asia	2 (10.5)	0
Europe	9 (47.4)	0
North America	3 (15.8)	0
South America	1 (5.3)	0
Base country *(*income group*)*		
High-income	11 (57.9)	0
Low- and middle-income	12 (63.2)	18 (100)

Note: most categories are non-exclusive (eg, participants may follow more than one profession, in more than one location).

N/A, Not Applicable.

**Figure 1:**
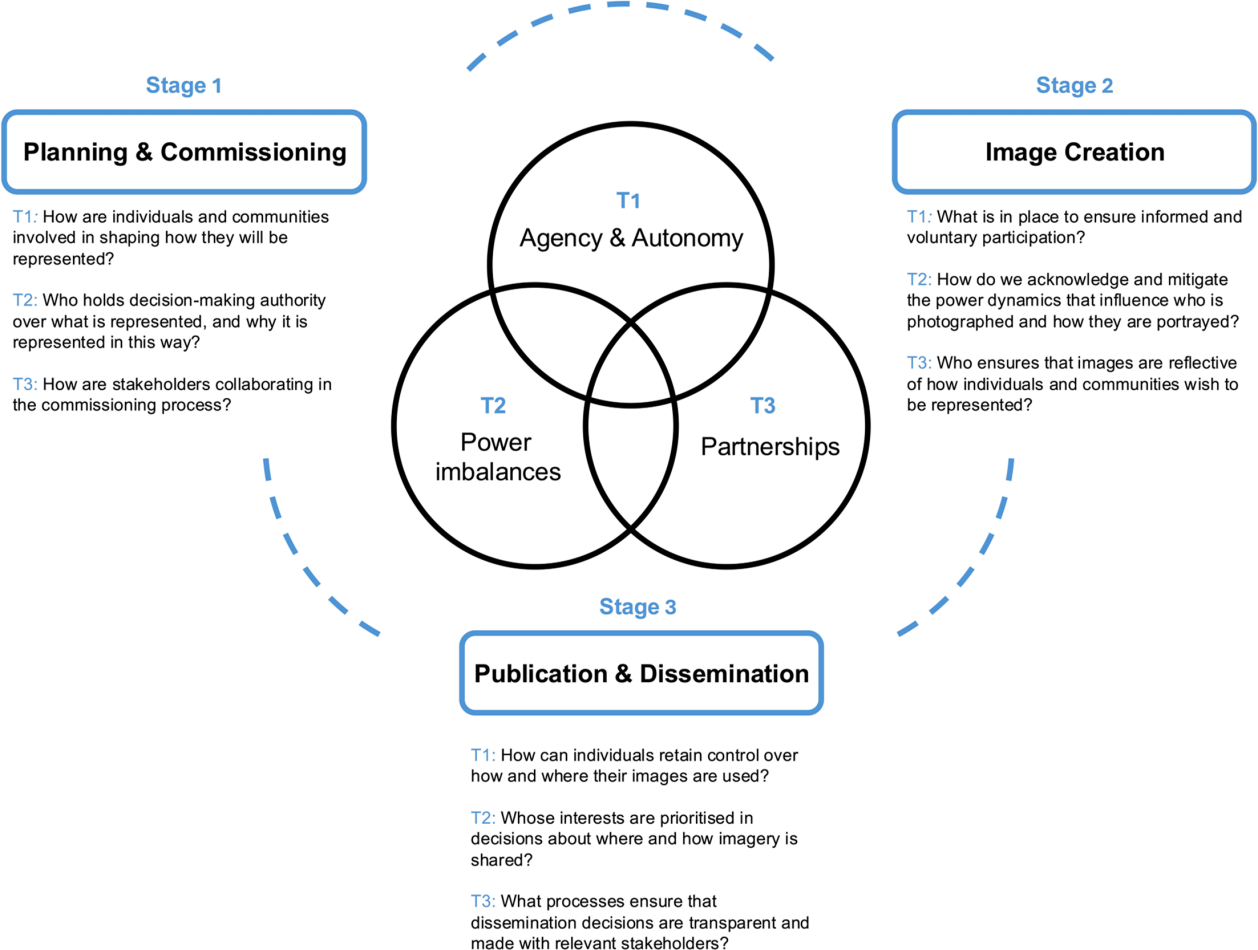
Summary of themes from interviews and learner survey responses (Theme 1 (T1): agency and autonomy; Theme 2 (T2): power imbalances; Theme 3 (T3): partnerships) across the three stages of imagery production (planning and commissioning; image creation; publication and dissemination).

Three key themes emerged from inductive thematic analysis of interview and survey data (see online supplemental table S1 for additional survey data). Agency and autonomy referring to the extent to which individuals and communities have meaningful control over how they are represented, including participation in decisions about image framing and use, free from manipulation or coercion. Power imbalances (inequity) regarding the unequal distributions of resources and influence between those who create and disseminate imagery and those who are represented, which may be reinforced through imagery itself. Collaborative partnerships among stakeholders involved in commissioning, producing, publishing and being represented in Global Health imagery. The data suggest that each of these three broad themes should be considered throughout the image creation and dissemination process. To be most practically relevant, results are discussed in relation to the following processes: planning and commissioning, the act of photography or image creation, and publication and dissemination of the images (figure 1). This is followed by a summary of practical recommendations table 2.

**Table 2:** Practical recommendations for ethical and equitable imagery in global health

Stage of imagery practice	Stakeholder group	Recommendations
Planning and commissioning	Commissioning and institutional actors	Develop ethical imagery policies and reflexive training addressing positionality, power, representation, dignity and narrative integrity. Use collaborative briefing processes involving image-makers and community partners. Avoid commissioning imagery based on stereotyped or deficit-focused narratives.
	Image-makers and researchers	Reflect on personal and institutional positionality, intended narratives and existing power imbalances before beginning projects. Engage local expertise and prioritise contextual understanding and safeguarding.
	Communities and participants	Participate in co-design and consultation processes regarding representation, storytelling priorities and culturally appropriate safeguards.
	Ethical oversight and governance bodies	Include consideration of imagery ethics, dissemination, safeguarding and future image use within ethical review processes, particularly when involving vulnerable populations.
Photography and image creation	Image-makers and researchers	Prioritise ongoing, context-sensitive and relational consent processes extending beyond written forms. Create opportunities for participants to express preferences regarding representation and storytelling. Engage in reflexive practice throughout image-making.
	Communities and participants	Enable participants to contribute actively to storytelling and decisions about representation. Ensure children and young people are supported through age-appropriate safeguarding and trusted adults where appropriate.
	Commissioning and institutional actors	Support inclusive representation while avoiding tokenistic inclusion. Allow sufficient time and resources for ethical relationship-building and consent practices.
Publication and dissemination	Commissioning and institutional actors	Ensure captions and accompanying narratives accurately contextualise imagery and minimise stereotyping or sensationalism. Support participant-informed dissemination and review processes where feasible.
	Image-makers and researchers	Maintain narrative integrity during editing and dissemination. Reflect on how platform, audience and context may alter interpretation of images.
	Ethical oversight and governance bodies	Encourage institutional accountability mechanisms for imagery governance, including guidance on digital circulation, AI manipulation, anonymisation and future image reuse.
	All stakeholders	Treat ethical imagery as an ongoing relational and reflexive practice rather than a static checklist, prioritising dignity, transparency, shared accountability and partnership throughout all stages of image-making.

Stakeholder groups included in this table: commissioning and institutional actors (eg, universities, NGOs, funders, publishers and media organisations); image-makers and researchers (eg, photographers, filmmakers, researchers and communications staff); communities and participants (eg, participants, young people, local organisations and community gatekeepers); and ethical oversight and governance bodies (eg, institutional review boards, ethics committees and institutional leadership). Importantly, these recommendations are meant to encourage reflective practice, not to serve as a checklist.

AI, artificial intelligence; NGO, non-governmental organisation.

### Planning and commissioning

Participants emphasised that ethical considerations begin at the earliest stages of planning and commissioning, particularly decisions about who holds authority, what is represented and why. Power dynamics were described as inherent to these decisions, which are often controlled by external actors such as NGOs, academics, funders or media organisations based in high-income settings. As a result, people represented in imagery, frequently from LMICs, often have limited influence over how they are portrayed (online supplemental table S2: T1Q1).

Participants stressed that imagery must accurately reflect people and communities in ways that align with their identities and perspectives. Misrepresentation was described as both ethically problematic and potentially harmful. One participant explained that photographing someone ‘in any situation that [they are] not comfortable (…) could even [make that person] feel humiliated’*,* further describing misrepresentation as ‘a form of psychological violence’ (online supplemental table S2: T1Q2). Such harms may extend beyond the moment of image capture, as vulnerable images can be widely disseminated, become permanent and later affect individuals’ safety, privacy or how their communities are perceived.

Planning is often initiated through briefs issued by commissioning entities (online supplemental table S2: T1Q3). Interviewees suggested that sharing briefs across teams, rather than with individuals alone, can support alignment with intended values (online supplemental table S2: T1Q4). However, participants cautioned that overplanning or overly prescriptive guidance can reinforce preconceived narratives and lead to artificially curated representations. Partnership with local stakeholders was therefore seen as essential, alongside offering image-makers space to learn from context and ‘human contact’ in deciding what should or should not be photographed (online supplemental table S2: T3Q1; T2Q1). Strict guidelines were described as potentially ‘intimidating’, making photographers ‘reluctant (…) to get involved’ (online supplemental table S2: T1Q5), and as sometimes promoting what is ‘socially acceptable more than what is like reality on the ground’ (online supplemental table S2: T1Q6), even while guidance can help set minimum standards (online supplemental table S2: T1Q7).

Participants also reflected on the role of the photographer. Image creation was described as reflecting the language of the artist (online supplemental table S2: T1Q8), with photographers often relying on intuition or ‘a second feeling’ to judge what is appropriate in a setting (online supplemental table S2: T1Q9). Some argued that a photographer should therefore not be an ‘outsider looking in’ who ‘[has not] really worked with the community*’* (online supplemental table S2: T 2Q1), while others felt that intention and values mattered more than origin (online supplemental table S4: T3Q1). Importantly, participants noted that even local image-makers may reproduce stereotypes, assuming this is what ‘will sell (…) to the western audience’ (online supplemental table S2: T2Q2). Good intentions alone were seen as insufficient if communities are excluded from decision-making (online supplemental table S2: T2Q3), with one participant highlighting that it is ‘the way’ in which a photo is taken that can make it ‘dignified or undignified’ (online supplemental table S2: T2Q4).

Partnership with residents and local organisations during commissioning was widely viewed as critical. Participants emphasised consultation, dialogue and ensuring communities have a leading voice in how images are created and used (online supplemental table S2: T3Q2). These discussions were underpinned by concerns about inequity, particularly the structural imbalance between people with cameras or high-income platforms and those whose lives are depicted, which risks reproducing exploitation or misrepresentation if unaddressed (online supplemental table S2: T2Q5, T3Q1).

### Photography and image creation

At the image creation stage, participants focused on how imagery is produced, who is represented and how representation occurs. Ethical image creation was consistently linked to safeguarding agency and autonomy, which participants viewed as foundational to informed consent and dignity. Informed consent was described as central to the integrity of the work (online supplemental table S2: T1Q10), including a commitment to ‘stop using people’s pictures to address something different [to what they agreed]’ (online supplemental table S4: T1Q1). Ensuring autonomy requires clear communication and confirming that individuals understand and actively agree to how they are portrayed (online supplemental table S2: T1Q11).

Participants emphasised that consent practices must be tailored to the preferences and culture of the represented community (online supplemental table S2: T1Q12; T1Q13). The framing of consent requires a shift from ‘information giving’ and a legal practice to the establishment of a professional relationship with the subject (online supplemental table S2: T3Q1, T3Q4). It should be seen as a dialogue, with safeguarding at its core, between people creating the image and people in the image—consent forms should only come at the very end (online supplemental table S2: T2Q6). Participants stressed that consent processes must extend beyond image capture to include discussion of future uses, particularly as identifiable imagery may place vulnerable individuals at risk (online supplemental table S2: T1Q19–20).

Working with children and young people introduced additional ethical complexity. Participants stressed the need for communication with parents or guardians, with children’s safety, privacy and best interests prioritised (online supplemental table S2: T1Q14). At the same time, children should not be excluded from discussions. Many are able to express discomfort or refusal, which should be respected (online supplemental table S4: T1Q2-3). One participant described holding consent discussions separately with children to create fewer intimidating spaces for their questions (online supplemental table S2: T1Q15).

Power imbalances were seen as unavoidable, requiring image-makers to exercise sensitivity and avoid ‘push[ing their] agenda’ (online supplemental table S2: T1Q16). One participant described their efforts to ‘make my presence known’ in public spaces to allow people time to refuse to be photographed or adjust their comfort, for example, by covering their face (online supplemental table S2: T1Q16).

The importance of inclusive representation was emphasised, including the need to ‘showcase people with different body shapes, sizes, ages and abilities’ (online supplemental table S4: T2Q1). Participants noted that those excluded from imagery are often also excluded from funding and decision-making, namely ‘people with disabilities, older people’. (online supplemental table S2: T2Q7). However, interviewees cautioned against tokenism, where marginalised groups are included superficially rather than through meaningful representation (online supplemental table S2: T2Q8-9). Employing more diverse staff was seen as one way to broaden perspectives (online supplemental table S2: T2Q10).

Involving subjects as active participants in image creation, and enabling ‘people [to] tell their stories on their own’ (online supplemental table S2: T1Q17), was viewed as central to equitable practice. Partnerships with local individuals and organisations could support this through co-developed consent processes, clear privacy boundaries and imagery that reflects community values (online supplemental table S2: T3Q5-6). As one participant explained, ‘true representation would be [achieved] when it comes from the people [who] were actually the subjects of the issue (…) rather than us talking on behalf of them’ (online supplemental table S2: T3Q3).

Participants also highlighted that inequities are embedded in the act of photography itself. Cameras were described as symbols of authority and wealth (online supplemental table S2: T1Q16; T2Q11), particularly salient when working with patients or communities in crisis who may feel unable to refuse being photographed. Such imbalances can undermine consent, as agreement under unequal conditions may not be fully voluntary (online supplemental table S2: T2Q12–13).

### Publication and dissemination

At the dissemination stage, participants focused on where imagery is shared and the reasoning behind these choices. Individuals should have a voice in decisions about how and where their images are used, including understanding the platforms and scale at which images may be disseminated and being able to consent or decline on that basis (online supplemental table S2: T1Q18). The near-impossibility of controlling online circulation was seen as something that must be clearly communicated (online supplemental table S2: T1Q21–22). Furthermore, concerns were raised that institutional priorities for wide dissemination often override individuals’ ability to control their image and maintain their privacy (online supplemental table S2: T2Q13-14).

Photographers also expressed a desire to be ‘active participants’ in decisions about the subsequent use of their work, including ensuring ‘that [artists] are consulted’ should there be a wish to use their work in ‘a context outside of the original agreement’ (online supplemental table S2: T3Q7). Participants also argued that individuals should be able to retract consent, as circumstances or feelings about image use may change (online supplemental table S2: T1Q18), but recognised the practical challenges of doing this (online supplemental table S2: T2Q15).

Financial incentives were seen to drive image selection, with stereotypical or harmful portrayals frequently favoured for perceived emotional impact and fundraising potential (online supplemental table S2: T2Q2). This practice was seen as reinforcing reductive narratives and perpetuating inequity, as the value of an image came to be less about the dignity of those represented and more about its perceived fundraising potential (online supplemental table S4: T1 Q4).

Participants highlighted the role of captions and narratives. These tools shape interpretation and can either reinforce or challenge harmful stereotypes (online supplemental table S2: T2Q16). Therefore, using images only in their originally intended context was emphasised, as altering surrounding narratives would likely alter the message conveyed by the image (online supplemental table S2: T1Q23-24). Another participant highlighted that the way images are interpreted will *‘*differ from context to context’. For example, an image considered ‘problematic’ in a ‘medical context’ might instead be placed ‘in a gallery’ with the intent of ‘engaging [the public] in a discourse of criticality’ (online supplemental table S2: T1Q25).

Collaborative review processes, including shared decisions on captions, layout and ‘final approval[s]’, were proposed as ways to mitigate inequity and reinforce agency (online supplemental table S2: T3Q8-9). While participants acknowledged practical challenges, such partnerships were viewed as important steps toward more ethical dissemination, particularly if community members are involved. Specifically, reviewing imagery prior to publication through advisory or review groups was suggested to identify ethical concerns and ensure respectful representation (online supplemental table S2: T3Q10). Determining what is ‘relevant or not’ was described as inherently difficult, as different people will have ‘their own priority’, reinforcing the need for diverse perspectives and collaboration (online supplemental table S2: T3Q11).

### Reflecting on a ‘framework’: merits and limitations

Participants reflected on the practicality of the framework proposed by Charani *et al* (2023), identifying three interrelated themes: the need for training and education; shared accountability and institutional responsibility; and the balance between generalisable principles and context-specific guidance. While the framework was seen as a valuable tool for structuring ethical decision-making around imagery, participants emphasised that its use depends on adequate training, leadership support and organisational resourcing (online supplemental table S3: T1Q1, T1Q2, T2Q1). Its emphasis on collective accountability was seen as a strength, recognising the need for collaboration among image-makers, organisations and communities, supported by transparent decision-making (online supplemental table S3: T2Q2). Views differed on the level of specificity required, with some favouring organisation-specific guidance to reduce ambiguity, while others appreciated the framework’s flexibility and creative freedom across contexts (online supplemental table S3: T3Q1). Engaging with guidelines was seen as a catalyst for reflection and cultural change, extending beyond the framework as a static set of rules (online supplemental table S3: T1Q3).

Among young image-makers enrolled in Eh!woza programmes, most found the guidelines easy to understand and believed they could support more ethical image-making practices (online supplemental material).

Stakeholder groups included in this table: commissioning and institutional actors (eg, universities, NGOs, funders, publishers and media organisations); image-makers and researchers (eg, photographers, filmmakers, researchers and communications staff); communities and participants (eg, participants, young people, local organisations and community gatekeepers); and ethical oversight and governance bodies (eg, institutional review boards, ethics committees and institutional leadership). Importantly, these recommendations are meant to encourage reflective practice, not to serve as a checklist.

## Discussion

Three key themes emerged from the analysis of interview and survey data. One key theme centred around agency and autonomy. These themes are defined as the extent to which individuals and communities have meaningful choice and control over how they are represented, and includes participation in decisions about image framing and use, free from manipulation or coercion. Power imbalances and inequity related to the unequal distribution of control, resources and influence between those who create and disseminate imagery and those who are represented were evident in the data. The role of partnerships and how they could be established equitably also emerged as an important issue in image-making.

Our findings demonstrate that ethical challenges arise at all stages of imagery practice, from planning and commissioning to image creation and dissemination. Power imbalances were consistently described as shaping these encounters, particularly in contexts involving vulnerable populations. During photography, unequal power relations may limit individuals’ ability to refuse participation, placing autonomy and agency at the core of preserving dignity. These findings align with Susan Sontag’s critique of photography as a non-neutral practice embedded in relations of power,^20 21^ and reinforce the ethical responsibility of photographers to exercise restraint when subjects are uncomfortable.

These tensions may become particularly acute in humanitarian emergencies or conflict settings, where opportunities for prolonged consent discussions or collaborative review may be constrained by urgency or immediate threats to life. Yet imagery produced in such circumstances may play a critical role in documenting abuses, mobilising humanitarian support, influencing policy and preserving historical memory. Nevertheless, principles such as dignity, safeguarding, minimising harm and informed consent remain important, including through approaches such as anonymisation, careful framing and limiting identifiable imagery where possible. Ethical frameworks for Global Health imagery should therefore support context-sensitive judgement rather than function as rigid rules, while continuing to emphasise accountability and the protection of those represented.

Interestingly, autonomy was also seen to apply to artists themselves.^22^ While organisational briefs can support alignment with project aims, they often reflect externally defined notions of relevance shaped by high-income settings,^4 9^ which risks producing imagery disconnected from local realities and lived experience.^23^ Participants highlighted the need to balance ethical guidance with creative freedom through dialogue and partnership rather than rigid prescription.

Power dynamics were viewed as particularly acute when working with children and young people, who may experience heightened pressure to consent due to age, social hierarchies or dependence on adults.^24^ Ethical consent therefore requires approaches that are developmentally appropriate, context-dependent and relational, involving support networks, parents and guardians while prioritising children’s own expressions of comfort or refusal.^25^ Where discussions are held separately with children to support autonomy or reduce intimidation, safeguarding remains essential, including the presence of a trusted adult or appropriate support person identified in accordance with the local context and best interests of the child.

At the dissemination stage, organisational agendas, financial incentives and the unpredictability of digital circulation were seen to further undermine agency and consent. Participants emphasised a shift away from extractive practices toward partnership-based approaches, in which individuals represented in imagery are meaningfully involved in decisions and regarded as collaborators rather than subjects. Photographers similarly expressed a desire for stronger partnerships with commissioners, as they, too, experience a loss of control and autonomy over their work once disseminated.

These findings resonate with broader critiques of power asymmetries in Global Health, in which narratives are often shaped by institutions and actors distant from the contexts they represent.^26 27^ Decisions about representation, therefore, constitute sites of political and ethical contestation, determining whose voices are amplified and whose interests are served.^4 5 9 10^ The emphasis on autonomy in image-making aligns with wider Global Health debates that call for prioritising the voices of those most affected, both in health practice and representation.^14 15 27^

Overall, participants viewed the framework as a valuable tool for structuring ethical reflection. Its flexibility was seen as enabling application across diverse contexts, but also as a potential limitation if not supported by training, leadership commitment and institutional accountability. Some participants expressed concern that without such support, engagement with the framework may remain superficial. These perspectives reflect an inherent tension between providing guidance that is specific enough to inform practice while remaining adaptable to artistic and contextual variation.

Emerging technologies further complicate this challenge. Advances in artificial intelligence allow images to be generated or altered with increasing realism, raising questions about authenticity, bias and consent. While artificial intelligence-generated imagery has been proposed as a means of protecting privacy,^28^ concerns persist regarding manipulation, fabrication and the reproduction of existing stereotypes and sensationalised depictions of poverty and saviourism, embedded in existing training datasets.^4 9 29^ These developments blur boundaries between documentation and creation, undermine trust in visual storytelling,^30^ and risk profound loss of agency over representation, reinforcing the need for transparency, labelling and accountability.

This study has limitations. The findings reflect a relatively small and self-selected group of participants and may not capture the full diversity of perspectives across contexts. Views of commissioning bodies and funders, who are key actors in shaping imagery practices, were not directly captured and warrant further investigation. Additionally, the rapidly evolving technological landscape means that ethical frameworks for imagery will require ongoing revision to remain relevant. Lastly, the interpretation of findings is also shaped by the positionality and experiences of the multidisciplinary research team, working across the UK and South Africa and across academic, healthcare and art-based disciplines. Ongoing reflexive discussion, comparison across diverse participants and collaboration with Eh!woza enabled better contextual interpretation and rigour of the analysis.

## Conclusion

In conclusion, this study underscores the need for more ethical, inclusive and context-sensitive approaches to imagery in Global Health. The framework discussed provides a foundation for navigating ethical challenges across all stages of imagery practice—planning, creation and dissemination. However, the framework’s effectiveness depends on meaningful training, institutional commitment and sustained engagement with communities, particularly vulnerable groups such as children and young people. Ethical imagery cannot be reduced to technical guidelines alone; it must be embedded within broader cultural and organisational shifts that prioritise dignity, accountability and authenticity. Ultimately, ethical practice requires ongoing collaboration between institutions, artists and communities to ensure that imagery not only informs but empowers, respecting those represented and maintaining integrity in the stories told.

## Supplementary material

10.1136/bmjgh-2026-024519 online supplemental file 1

10.1136/bmjgh-2026-024519 online supplemental file 2

10.1136/bmjgh-2026-024519 online supplemental file 3

## Data Availability

No data are available.
