# Supplementary material for: Equitable imagery in global health: a qualitative study examining how to create agency, share power and build partnership
Source: BMJ Glob Health. 2026 Jul 13;11(7):e024519. doi: 10.1136/bmjgh-2026-024519 (PMC13365758; doi:10.1136/bmjgh-2026-024519)
Supplement: online supplemental file 1 [file bmjgh-11-7-s001.docx]

**Supplementary Material: Results**

**
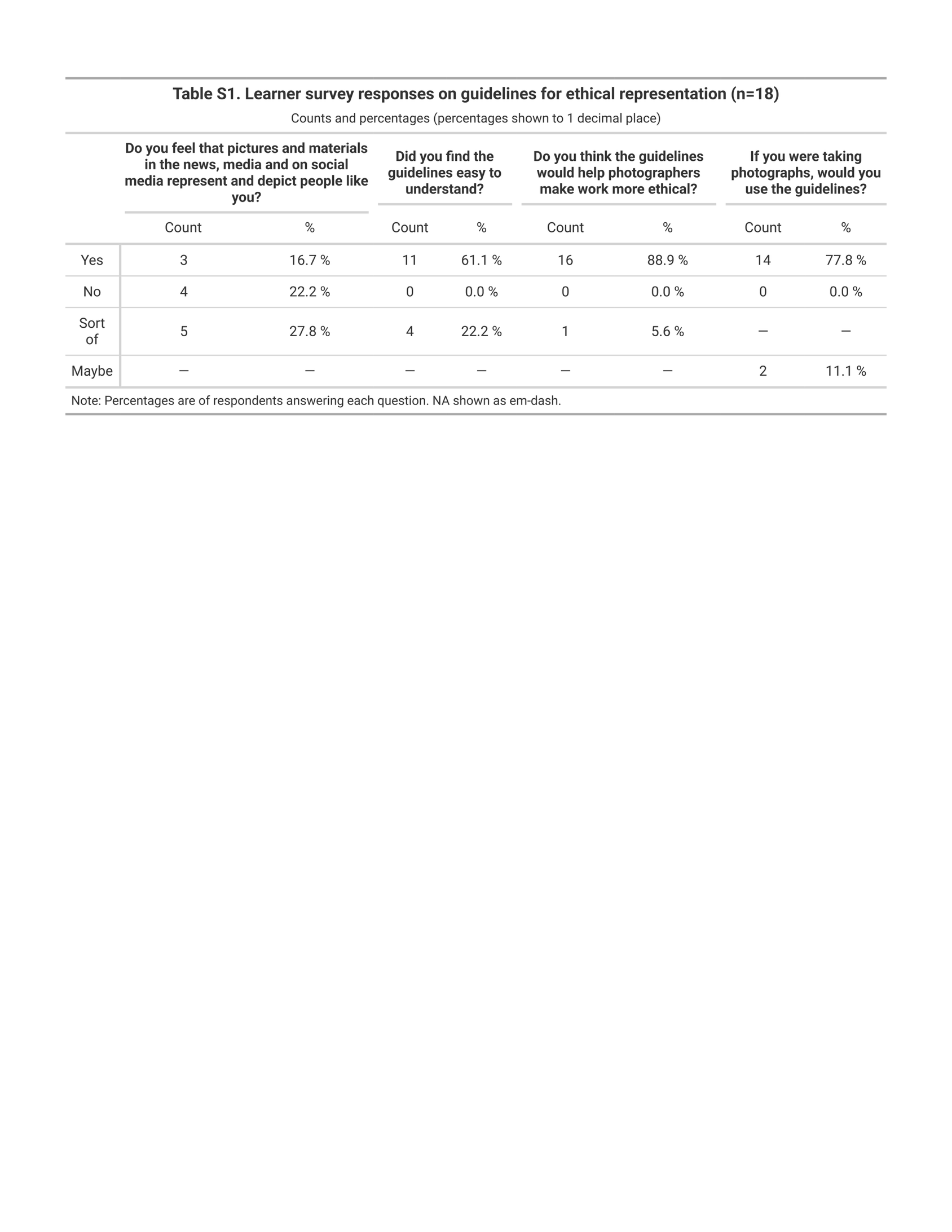
Table S1: Learner survey responses on guidelines for ethical representation (n=18)**

**Supplementary quotes tables**

**Table S2**: Quotes for each of the three principal themes derived from interviews (n=19)

| **Theme** | **Quote Number** | **Quote** |
| --- | --- | --- |
| ***1: Agency and Autonomy*** | 1 | *"I might be defining myself as a migrant young woman, and I wouldn't have an issue with my picture being used in a context that I advocate for. (…) But someone else might not be happy to be publicly defined as a migrant. They might not even be a migrant." (Participant 13)* |
|  | 2 | *"It can be even a form of violence: if you present a picture of someone in a vulnerable situation without their consent, I mean, if you think about yourself, if you're, you know, sick, or just in any situation that you're not comfortable, and then someone takes a picture, and that picture is published, you could even feel humiliated (…). It can be a form of psychological violence on the people that are represented incorrectly."* (Participant 6) |
|  | 3 | *"When we're talking about the context, (…) this is the kind of thing that is really useful if it's included in a brief from an organisation. Because in practice, quite a lot of photographers will be doing different kinds of work, and (…) there will be different requirements and different standards involved (…). So, it's really important for organisations to be really clear about what they want, and the standards that they want their photographers to meet."* (Participant 6) |
|  | 4 | *"But there is (…) an information gathering element (…) to the photographic process, which sometimes is done by a comms person. Sometimes it's done by a local, you know, a member of the local team, and sometimes it's done by the photographer. And that is another area where ethical standards can be really enhanced with a clear brief."* (Participant 6) |
|  | 5 | *“Photographers might become reluctant to photograph subjects or to get involved in certain subject matter, if they feel like they can't, you know, overcome the complexity of the guidelines. So I think (…) explaining it in a way that makes it really understandable but also accessible in terms of how to implement them, then they will be less intimidating.”* (Participant 16) |
|  | 6 | “*I think that's also where you start (…) trying to adapt to what's, like, socially acceptable more than what is like reality on the ground.”* (Participant 4) |
|  | 7 | *“This is the kind of thing that is really useful if it's included in a brief from an organisation. Because in practice, quite a lot of photographers will be doing different kinds of work, and (…) there will be different requirements and different standards involved. So for example, for a news photographer, the standards of consent are very different to a documentary, so it's really important for organisations to be really clear about what they want, and the standards that they want their photographers to meet.”* (Participant 7) |
|  | 8 | *“I didn't realise that I just keep on revisiting my childhood, and the work that I make are is like extensions, or like extensions of myself, really.”* (Participant 15) |
|  | 9 | *"I just have a second feeling or know what I need for the images to look like? I don't know. It just really does come from somewhere else."* (Participant 15) |
|  | 10 | *"You really can't have anything that shows integrity without consent."* (Participant 8) |
|  | 11 | *"I think [consent is] super important. You're using their image and likeness (…) in your work, and therefore, they should have the right to opt in or opt out of that. And so, yeah, I think [it's] really [about] building an understanding of how their image will be used. And what it will be used for (…) particularly when we're talking about vulnerable or stigmatised groups."* (Participant 1) |
|  | 12 | *"For photographs, I mean, (…) depending on where you go, (…) sometimes (…) culturally (…) a mother (…) will have like, far less say over consent (…) as opposed to the father. And sometimes the father has even less consent than the chief. And yeah, and if you intervene in the wrong tier, you could (…) actually cause trouble (…)."* (Participant 5) |
|  | 13 | ***"****So just having a lot of humbleness when you go into a situation in a context that you don't know, and trying to inform yourself to scope as much as possible before coming up with an idea of how to establish consent."* (Participant 6) |
|  | 14 | *“I think, particularly because children are young (…) they are in a position of vulnerability. It's not always evident that proper informed consent has been obtained, where is the parent in this scenario? And so like, I think, [children are] easier to be taken advantage of, than perhaps an adult who might be like, ‘Excuse me, why, why are you doing this?’. And so I think a framework is particularly important in safeguarding the rights of children here.”* (Participant 1) |
|  | 15 | *"So, children should be involved in the consent process (…) And what I like to do if you're (…) going to be in a [photography session] where you're photographing several children, is to have the children together, because then it's less intimidating. Because the more brave and confident ones will ask questions, and then the shy ones will be like, ‘Oh, yeah, I wanted to ask that too.’ (…) But yeah, I think involve kids. They often will ask the more difficult and challenging questions."* (Participant 10) |
|  | 16 | *"You try to read the room. (…) In general, you can get a good feeling for if someone is comfortable or uncomfortable with you being there. I make a point of making my presence known (…). So when a tall white dude with an expensive camera walks in, they know what he's doing there. And I don't try to hide that. (…) If I'm going into (…) a public place, like say, like a waiting room in a hospital or something like that. I'll ask whoever is, you know, the head of the ward or department, or [whoever] it is, to make (…) a general announcement saying who I am, where I'm from, you know, what's going on? Yeah, and announce that (…) if you're uncomfortable, please, you know, either leave or cover your face. Or if I approach you, just give me like a, (…) a ‘no, no, thanks’ kind of thing. And that's perfectly fine to do. Yeah, (…) and then I'll generally wait like 10-15 minutes and walk in and then suddenly move around. (…) If they [are] feeling (…) comfortable having you around, then (…) you're aware of that. As soon as you see someone flinching or trying to turn around, (…) I don't try to fight that. (…) I don't push my agenda overly"* (Participant 5) |
|  | 17 | *"But with what I do, I let the people tell their stories on their own, with their permission."* (Participant 14) |
|  | 18 | *"Like [really] making sure that you have (…) consent, (…) not just (…) before (…), [but] also after. (…) What a person says, [or] how they're portrayed, [should not necessarily be] a thing that lasts forever, you know. So, we often have [situations] that five years down the line, someone comes back [with] stuff circulating, saying, ‘I don't like the way I'm being portrayed now.’ And we go, ‘That's absolutely fine. Let's remove (…) any trace of this.’ So it also has a bit of a lifespan, and it evolves as people change and as the world changes and things like that."* (Participant 11) |
|  | 19 | *“They [participants] should have also been, like, made aware of how that image is going to be used and what kind of archive.”* (Participant 17) |
|  | 20 | *"Taking a photo of someone on the street might look nice or even powerful, but that person might not be safe if that image is shared widely, especially online or on social media."* (Participant 13) |
|  | 21 | *"For me, it's explaining to them that a final product will go online. So, there's a possibility that it can trend. There's a possibility that it can go on TV, you know, like, and then I tell them, like, all the social media platforms that we're using and which ones (…) are likely to trend, you know, and also explain that this might be shown like around the world, you know."* (Participant 14) |
|  | 22 | *"I think the, sadly, with the structure of the industry is that with the more commission jobs I'm doing these days (…) generally people are asking you to hand over imagery, and then they use them, you know, as they wish. So, you as the photographer, (…) are generally quite removed from the final products (…)."* (Participant 5) |
|  | 23 | *"And I think that that's something that's so integral to the art world in terms of how (…) you can't (…) change the way that (…) somebody's painting a show. You can't crop somebody's image on social media because it tells a different story. And so yeah, I think that's a big part of it."* (Participant 17) |
|  | 24 | *"And (…) to insist that if, if there's a context outside of the original agreement, (…) that they are consulted".* (Participant 17) |
|  | 25 | *“I also think that (…) the ethics of (…) using an image [can] differ from context to context. So in a medical context, in the gallery context, those conditions are going to be very different. Because for instance, in a gallery context, (…) an image can be seen as engaging in a discourse of criticality. So you could have a public, problematic image that's there intentionally to (…) promote a kind of engagement in a certain discourse. Whereas that's not going to be the case for the medical context there.”* (Participant 16) |
| ***2: Power Imbalances (inequity)*** | 1 | *"I think [the framework] is just a respectful way of image making, because some people don't think about it, especially if you’re an outsider looking in and haven't really worked with the community (…)."* (Participant 15) |
|  | 2 | *“So they will just think, what will sell a good picture to the western audience. And they won't think what story am I trying to tell? The story they're trying to tell really is, there's some poor people that need our money. So they will always want to take those kinds of pictures. I think that's a really challenging area”* (Participant 2) |
|  | 3 | *"When you advocate for a group of people, especially in global health, it often comes from a place of goodwill—we want to support and speak up for people impacted by health inequalities. But sometimes, because of that good intention, people think it gives them a kind of right to use whatever image or story is available to get the message across."* (Participant 13) |
|  | 4 | *"You can take the same photo of a scene, and it can be dignified or undignified, depending on the way that it's taken."* (Participant 17) |
|  | 5 | *"Global health advocacy is (…) mostly coming from (…) academic people in high-income countries nowadays. And I think there's an aspect of colonial culture in there."* (Participant 13) |
|  | 6 | *"And there's [also] that a form is the worst way possible to do it. So, the form is [there so] that if you need to get a form [signed] to protect yourself legally, great. But you do that at the very end of a process, which is about conversation."* (Participant 10) |
|  | 7 | *"It's so often [that] there's not a focus on those who are even more vulnerable and forgotten, that are not necessarily pulling at the heartstrings so much. And I'm talking about people with disabilities, older people."* (Participant 3) |
|  | 8 | *"Fundamentally, if you're [at] an organisation, where (…) a particular team [is] being photographed, there really [are usually] only men in positions of power, which, obviously (…), is an issue, but fundamentally, that's how it is. You have to photograph what is there."* (Participant 1) |
|  | 9 | *"For example, (…) you will see people just brought in to balance the photo. (…) When I see people brought in just to make the photo, look, you know, I wouldn't want to be in that position. I would find that quite demeaning. And so, I think you have to represent reality, and then say, ‘This is a problem’."* (Participant 2) |
|  | 10 | *"One of the things that we've majorly done is, first of all, I've hired a diverse staff (…). I think the easiest way to fix a lot of these problems is just to hire (…) a better cross-section of humanity. So you have more people just like illustrating their natural experience."* (Participant 8) |
|  | 11 | *“But, no, but we're all particularly within healthcare and all that you can, you can see when someone's in an uncomfortable situation, and you put the camera down at that point.”* (Participant 5) |
|  | 12 | *“When you're dealing with vulnerable peoples in a situation, and you're going in with an organisation that is providing aid for those people, I think, to be honest, they've never actually got a voice over whether, you know, they can actually consent or not. Because, I mean, you go (…) to someone who's starving and you holding a loaf of bread and one hand and you and you say ‘Hey, can I take your photograph’, they're probably going to say, ‘sure, you can’, so that they can make sure they can get that loaf of bread (…) So even if they are consenting and all that I don't believe (…) they're in a position to truly consent. (…) I'm troubled with (…) these discussions of like, dignity and privacy and all that, it's far deeper than just (…) asking for permission.”* (Participant 5) |
|  | 13 | *“I think there's a policy element here, which is what is acceptable for the organisation, because, you know, there are some people who believe that first of all, informed consent - is it truly informed consent, if you're talking about vulnerable people, or people in poverty or people in conflict? It's not possible. You know, people who are reliant on an organisation, when somebody from that organisation comes along and says, ‘Hey, would you mind being in this photograph?’ (…) the power imbalance is so, it's so stark that it's really hard for them to say no.”* (Participant 7) |
|  | 14 | *"But it's also in the [age] of social media, very difficult once the image is in the digital realm. So now, our consent forms make that clear, and we say we will do our best to respect you, but you also have to go into this knowing (…) that (…) because of how images proliferate the digital paradigm (…) you don't have control over how [they spread]."* (Participant 16) |
|  | 15 | *“Yep, great. I'll give consent. There's a lot of pressure and power dynamics operating in that moment. They feel (…) like, it's hard to say no. But also maybe [the image subjects] feel great about it, and they're okay. But then they go home, or they're having a cup of tea with their mate the next day, and the mate goes, ‘Alright, what happened yesterday?’, [they reply] ‘Oh, I got filmed’. And the mate starts asking questions, and they go, ‘Ah, I didn't ask those questions’. And then they feel worried, and sometimes even really distressed, and maybe just a bit curious, and that they haven't had time to have that conversation again. And then [the photographer is] like, ‘Oh, I gave [the image subject] a phone number they could call’ but for many reasons, people often don't call those phone numbers, [because of], power, or logistics, or whatever.”* (Participant 10) |
|  | 16 | *"I contextualise that image, (…) maybe [if] it stands by itself, it might be easily seen as unethical and stuff. So there's a lot of important work that goes [into] editing or postproduction and making sure (…) that the narrative (…) is not heavy on, (…) what might be seen as a negative, (…) unethical portrayal."* (Participant 18) |
| ***3: Partnerships*** | 1 | *"And it's (…) about human contact and almost common sense that (…) you can tell. I think it's even with cultural differences, you can often tell when someone is uncomfortable, or when someone is not really, you know, into the idea of being in a photograph."* (Participant 6) |
|  | 2 | *“The big learning lesson for me is [that] it's really about who decides (…). Our cultures are different, you know, our ways of thinking are different. And people's situations are different (…), so I think if there [were] one thing I would add [is] that (…) this is not a decision made by (…) the visiting expat humanitarian or doctor or whatever. This is something that really should be led by the community."* (Participant 2) |
|  | 3 | *"My understanding is just [that] true representation would be [achieved] when it comes from the people [who] were actually the subjects of the issue (…) rather than us talking on behalf of them."* (Participant 13) |
|  | 4 | *"First, if possible, you have to establish some relationship with the people that you want to represent. So first, before taking any picture, there has to be a form of, you know, relation, and possibly asking if they want to be represented, and how [they want to be represented], I mean, and also (…) being very open about what you want from this picture."* (Participant 6) |
|  | 5 | *"You can't think about it as being a solitary pursuit. You can't think of yourself as moving [separate] from people. And I think that's one of the things that has been emphasised in documentary photography, is that (…) it only works (…) when you treat it as a collaboration and an engagement. Because that's how you get the interesting stories that [are] mutually beneficial, that's how you get the insight that makes something interesting. (…) I'm basically treating people as humans and not as (…) alien subjects that need to be (…) observed."* (Participant 17) |
|  | 6 | *"(…) I think, yeah, let's keep like, representing people in a good way, in a respectful way, making people more comfortable sharing their stories, making sure that we don't tell people's stories [for them] and [instead] let people tell their stories on their own (…)"* (Participant 14) |
|  | 7 | *“I think that's also an important part about photography and the way that we treat artists (…) we [should] allow them to be active participants in framing how they want their work to be shown (…) and to insist that if there's a context outside of the original agreement, that they are consulted”* (Participant 17) |
|  | 8 | *“I assume that picture is described, in a way with words, maybe just choosing the right words. And if you can do just getting those people read those words, as well, and making sure that those people actually know what's in what sort of concept that we are using those pictures, and what they are going to represent”* (Participant 13) |
|  | 9 | *“When the image is in its final form [and] ready to use, are we getting a final approval from [image subjects]? I think would already be something that would (…) [help] uphold the dignity and privacy [of image subjects].”* (Participant 12) |
|  | 10 | *"I think it does [need to] have some kind of a review system (…) almost like a peer review kind of system (…) sometimes people use harmful language without realising it. And it's not that they tried to be harmful. (…) it's [time] to have a system in place where someone can say, ‘This should be flagged’ (…), a place where someone could say, like, ‘I would like to submit my work for a review and see if there's any red flags(…)’"* (Participant 11) |
|  | 11 | *"It also becomes a very subjective topic for people deciding what is relevant or not – [Global Health] is quite diverse and everybody has their own priority – and when it's about coming to a consensus, it's hard."* (Participant 12) |

**Table S3**: Quotes for each of the themes about the ‘Framework in Practice’, derived from interviews (n=19)

| **Theme** | **Quote Number** | **Quote** |
| --- | --- | --- |
| **1: Need for Training and Education** | 1 | *"[A framework] also only works if you lay on a foundation of knowledge. So you have to spend time with organisations, training them and helping them understand and discussing this continually in order for these frameworks to land (…)"* (Participant 10) |
|  | 2 | *"I think [if] the guidelines were accepted, [it is also about] finding a few people to help champion them. And then it's the implementation of the guideline (…), which involves (…) continuously reminding people and doing some teaching around it, and so on"* (Participant 2) |
|  | 3 | *"I also think that the process of creating the guidelines is that education process (…) it's all very well providing guidelines like this, but if people haven't realised that there's a problem, and they need to change, these guidelines are not very helpful."* (Participant 10) |
| **2: Importance of Accountability & Collective responsibility** | 1 | *“And ultimately, what you get a lot of this kind of guidance, or people are more junior level, trying really hard to enact change. But if it doesn't have CEO level support, it's like dead in the water.”* (Participant 10) |
|  | 2 | *"I think we all have a responsibility [to] the people they are trying to advocate for."* (Participant 13). |
| **3: The balance between Generalisability vs Specificity of Guidelines** | 1 | *"And I think (…) creating more (…) frameworks that are more specific to different regions and conditions, (…) I think would be more beneficial instead of having (…) blanket rules."* (Participant 5) |

**Table S4**: Quotes for each of the themes about the ‘Framework in Practice’, derived from Learner questionnaires (n=18)

| **Theme** | **Quote Number** | **Quote** |
| --- | --- | --- |
| ***1: Agency and Autonomy*** | 1 | “Media should stop using pictures of people without their concern and they should stop using people's pictures to address something different.” (Learner 10) |
|  | 2 | “I would not like my health to be shown on media because it is private and a person must ask for my permission before they publish my picture” (Learner 17) |
|  | 3 | “I'd want to be shown as a reliable, trustworthy of inclusive source of health info” (Learner 16) |
|  | 4 | “People should be shown in pictures on social in a way that promotes positive and realistic representations of health” (Learner 15) |
| ***2: Power Imbalances (inequity)*** | 1 | “Showcase people with different body shapes, sizes, ages and abilities” (Learner 9) |
| ***3: Partnerships*** | 1 | “Avoid racism and other forms of bias. People should be respected by the photographer.” (Learner 11) |
