# Supplementary material for: Equitable imagery in global health: a qualitative study examining how to create agency, share power and build partnership
Source: BMJ Glob Health. 2026 Jul 13;11(7):e024519. doi: 10.1136/bmjgh-2026-024519 (PMC13365758; doi:10.1136/bmjgh-2026-024519)
Supplement: online supplemental file 2 [file bmjgh-11-7-s002.docx]

**Supplementary Material: Interview Guide**

*The guide is divided into sections with approximate timings. The main questions are delineated in black text, with follow-ups for each in blue, as bullet points.*

**Part 1: General views and applicability of the framework [15min]**

Thinking about your daily work, how is this framework relevant to what you do?

- How or where do you use images of people in your line of work? (e.g. teaching, research, writing etc.)

What do you think of this framework?

- What – if any – is the value of introducing a framework when representing people in Global Health?
- How effective do you think the framework is?
  - Had you considered representation of people in Global Health to be problematic before you came across this framework?
- Who would benefit most from the use of this framework?
  - Thoughts specifically about children/adolescents?
- Where (or in what practical contexts) could it be applied?
- What do you think are the limitations of its use?

**Part 2: Critique of each section of framework and suggestions for improved applicability [25min]**

*I was now hoping to gather your views for each recommendation, or section, of the framework – including how they could be applied in practice, and anything you think it has missed.*

**2A: Relevance:** in the context of Global Health, how important is it to ensure relevance of the image to the topic?

- Throughout your work, how have you ensured that the images you use/take are ‘relevant’ to the work you are producing?
  - *For those who have not applied this in practice themselves:* how have you seen this being achieved?
- How is this different for children/young people (if at all)?
- What challenges did you face? How did you overcome them?

**2B: Dignity:** how important is it to respect the dignity and privacy of individuals represented in Global Health images?

- How have you ensured that the dignity of people and communities are respected through imagery used in your work?
  - *For those who have not applied this in practice themselves:* how have you seen this being achieved?
- How is this different for children/young people (if at all)?
- What challenges did you face? How did you overcome them?
  - *For those who have not applied this in practice themselves:* what challenges do you think may arise? How would you overcome them?

**2C: Consent:** how important is it to ensure informed consent when representing people in Global Health images?

- How have you managed to ensure informed consent from individuals participating in your work?
  - *For those who have not applied this in practice themselves:* how have you seen this being achieved?
- How is this different for children/young people (if at all)?
- What challenges did you face? How did you overcome them?
  - *For those who have not applied this in practice themselves:* what challenges do you think may arise? How would you overcome them?

**2D: Representation:** how important is it to ensure adequate representation of people and communities in Global health images?

- How have you thought of and managed to represent individuals and communities through your work?
  - *For those who have not applied this in practice themselves:* how have you seen this being achieved?
- How is this different for children/young people (if at all)?
- What challenges did you face? How did you overcome them?
  - *For those who have not applied this in practice themselves:* what challenges do you think may arise? How would you overcome them?

**2E: Equity & Justice:** In your opinion, does this framework promote equity in Global Health? If so, how? If not, what is it missing?

- What further principles or considerations do you think should be added to this framework?

**Part 3: The framework in the context of AI [15min]**

What experience do you have in developing, disseminating or reviewing AI-generated imagery (if any)?

How might AI affect the applicability of this framework?

How do you think this framework can adapt to encompass AI-generated imagery?

**Part 4: Conclusion [5min]**

Is there any other thought you would like to share regarding the framework or its applicability to your work in global health?

Do you have any questions for me?
