# Supplementary material for: Equitable imagery in global health: a qualitative study examining how to create agency, share power and build partnership
Source: BMJ Glob Health. 2026 Jul 13;11(7):e024519. doi: 10.1136/bmjgh-2026-024519 (PMC13365758; doi:10.1136/bmjgh-2026-024519)
Supplement: online supplemental file 3 [file bmjgh-11-7-s003.docx]

### BMJ Global Health Author Reflexivity Statement

Adapted from Morton, B., Vercueil, A., Masekela, R., Heinz, E., Reimer, L., Saleh, S., Kalinga, C., Seekles, M., Biccard, B., Chakaya, J., Abimbola, S., Obasi, A. and Oriyo, N. (2022), Consensus statement on measures to promote equitable authorship in the publication of research from international partnerships. Anaesthesia, 77: 264-276. <https://doi.org/10.1111/anae.15597>

| **Study conceptualisation** | |
| --- | --- |
| 1. How does this study address local research and policy priorities? | This study addresses ongoing discussions in global health regarding equity, power, and representation in visual communication. By examining perspectives from photographers, researchers, NGOs, and young participants in Khayelitsha, South Africa, the study explores how ethical image-making practices can better support dignity, agency, and equitable partnerships in global health communication. |
| 1. How were local researchers involved in study design? | The study was conceptualised collaboratively by authors based in the UK and South Africa. Researchers affiliated with the University of Cape Town and Eh!woza contributed to shaping the research questions, recruitment approach, and interpretation of findings to ensure contextual relevance. |
| **Research management** | |
| 1. How has funding been used to support the local research team(s)? | Funding supported researcher time and collaboration between partner institutions, as well as engagement with the Eh!woza programme that facilitated participation from young image-makers in Khayelitsha. |
| **Data acquisition and analysis** | |
| 1. How are research staff who conducted data collection acknowledged? | Interviews and initial analysis were led by AMCP. Surveys with young learners were led by AK and EY. Contributions from all authors are recognised through authorship. |
| 1. How have members of the research partnership been provided with access to study data? | All authors had access to anonymised transcripts and analysis materials through secure institutional data-sharing platforms. |
| 1. How were data used to develop analytical skills within the partnership? | Qualitative coding and interpretation were conducted collaboratively, with mentorship from senior authors. |
| **Data interpretation** | |
| 1. How have research partners collaborated in interpreting study data? | Findings were discussed iteratively among the multidisciplinary author team to incorporate perspectives from global health, clinical research, and community-based creative practice. |
| **Drafting and revising for intellectual content** | |
| 1. How were research partners supported to develop writing skills? | The manuscript was initially drafted by AMCP with guidance and iterative feedback from AK, followed by revisions and input from all co-authors. |
| 1. How will research products be shared to address local needs? | Findings will be disseminated through open-access publication and academic and educational networks, contributing to discussions on ethical imagery practices in global health. |
| **Authorship** | |
| 1. How is the leadership, contribution and ownership of this work by LMIC researchers recognised within the authorship? | Researchers based in South Africa contributed to study design, interpretation of findings, and manuscript development and are recognised through authorship. |
| 1. How have early career researchers across the partnership been included within the authorship team? | AMCP, an early-career researcher, led the conceptualisation, data collection, analysis, and initial manuscript drafting and is recognised through first authorship. |
| 1. How has gender balance been addressed within the authorship? | The authorship team reflects attention to gender diversity across contributors. |
| **Training** | |
| 1. How has the project contributed to training of LMIC researchers? | The project supported collaborative learning across the partnership, particularly in qualitative research and ethical reflection on imagery practices. |
| **Infrastructure** | |
| 1. How has the project contributed to improvements in local infrastructure? | Although the study did not involve infrastructure development, collaboration with Eh!woza strengthened partnerships between academic institutions and community-based creative programmes. |
| **Governance** | |
| 1. What safeguarding procedures were used to protect local study participants and researchers? | Ethical approval was obtained from the University of Cape Town Human Research Ethics Committee. Interviews were recorded, transcribed, anonymised, and recordings deleted after transcription. Youth survey responses were collected anonymously and data were stored on secure institutional servers. |
